# Incidence of Tooth Loss in Remote Indigenous Populations of the Amazon Region: A 13-Year Cohort Study Before and After Belo Monte Dam

**DOI:** 10.3390/ijerph22010128

**Published:** 2025-01-20

**Authors:** Renata Travassos da Rosa Moreira Bastos, Eduardo Oliveira da Costa, Lucca Sicilia, David Normando

**Affiliations:** 1 Post-Graduation Program of Dentistry, Department of Orthodontics, Faculty of Dentistry, Federal University of Pará, Belém 66075-110, PA, Brazil; rtbastos@ufpa.br; 2 Master’s Degree Program, Department of Orthodontics, University Center of Pará (CESUPA), Belém 66060-575, PA, Brazil; 3 Independent Researcher, Belém 66055-490, PA, Brazil; eduardooliveiradc@hotmail.com; 4 Faculty of Dentistry, Federal University of Pará, Belém 66075-110, PA, Brazil; lucca.brasileiro@ics.ufpa.br; 5 Department of Orthodontics, Faculty of Dentistry, Federal University of Pará, Belém 66075-110, PA, Brazil

**Keywords:** indigenous people, tooth loss, incidence, epidemiologic studies

## Abstract

Tooth loss among indigenous people in the Amazon emphasizes the need for culturally appropriate oral health interventions. The objective of this study was to analyze the incidence of tooth loss in two remote Amazon indigenous populations. This prospective cohort evaluated a total of 47 indigenous in the permanent dentition at T0 and thirteen years later (T1) from two villages, Arara-Laranjal (n = 28, mean age 16.1 and 29.9 years) and Assurini do Xingu (n = 19, mean age 15.9 and 29.5 years), of different ethnic groups. A multilevel Poisson regression model assessed the influence of village, sex, and age on tooth loss. At T0, the indigenous people had all their permanent teeth. Forty-two lost at least one tooth (89%), and a total of 172 teeth were lost at T1 at an incidence of 97% among females and 76% in males. There was no influence of ethnicity on tooth loss (*p* = 1.000). A lower risk of tooth loss was associated with male subjects (β = −0.50, *p* < 0.05) but not with age. In females (22/46.8%) and males (11/23.4%), the highest incidence of tooth loss was the lower second molars. The risk was higher among females, and there was no influence on age, village, or ethnicity. The second and first molars were the most affected teeth. These findings suggest an increase in tooth loss caused by close contact between indigenous and urban populations.

## 1. Introduction

Tooth loss is a common finding in the human population of underdeveloped countries due to the social and regional inequalities, particularly with regard to access to basic health services [1]. Poor oral hygiene and changes in dietary pattern, characterized by a processed and industrialized diet, are highly associated with the etiology of these losses [2,3,4].

The Arara and Assurini indigenous populations, located in the region of the Middle Valley of the Xingu River, state of Pará, Brazil, are semi-isolated communities in which the maintenance of subsistence activity comes from traditional dietary habits based on cassava, nuts, fish, meat of wild animals, sweet potatoes, yams and fruits [5]. Recent close contact with non-indigenous people facilitated access to processed foods and sugar-based drinks, which impacted their oral hygiene and led to a high incidence of dental caries [3,6]. This contact, potentiated by the construction of the Belo Monte Dam in the Xingu River, approximately 200 km away from the villages, from 2011 to 2019, followed by the operation in 2016, led to their introduction to the urban environment due to the arrival of thousands of people to the region which led to the displacement of the indigenous population [7,8]. This scenario certainly caused a series of social, cultural, economic, and environmental consequences for these people, as well as the arrival of industrialized sugar-based foods, furnished by visitors as a form of pleasing the affected communities [9].

Tooth loss represents an important clinical endpoint of oral health because it is an early indicator of the health–disease process and social inequality, especially in vulnerable groups, characterized by social marginalization and isolation, socioeconomic disadvantage, and limited access to healthcare [10,11]. Data related to indigenous health in Brazil reveals that tooth loss, dental caries, and periodontal disease are dental–pathological indicators that characterize the major oral health problems faced by Amazon indigenous populations of different ethnicities [12].

The loss of oral health inevitably creates a negative impact on general health and quality of life [3,6]. Given this scenario, this study aims to analyze the incidence of tooth loss of two indigenous populations belonging to different ethnic groups in two distinct periods, 13 years apart.

## 2. Materials and Methods

### 2.1. Ethics Statement

This study was approved by the Research Ethics Committee of the Institute of Health Sciences of the Federal University of Pará and by the National Ethical Committee (CONEP) for Health Sciences of Brazil (1.433.511) and is in accordance with The Code of Ethics of the World Medical Association (Declaration of Helsinki) for experiments involving humans. The indigenous population was informed about the objectives of the study and authorized their participation by signing the free and informed consent form in accordance with the Brazilian National Health Council, resolution 466/12. For the indigenous who did not know how to read or sign, verbal consent was obtained through an audio recording. The permission to enter the indigenous villages and collect data was obtained by FUNAI (National Indigenous Foundation), after prior contact with the Xingu indigenous leaders.

### 2.2. Study Design, Participants, and Eligibility Criteria

This was a prospective cohort study with follow-up after 13 years, and the Strengthening the Reporting of Observational Studies in Epidemiology (STROBE) guidelines for prospective cohort studies were followed [13]. The convenience sampling was the same method that had already been used in 2009 in previously published studies carried out in indigenous populations located in the region of the Medium Valley of the Xingu River, State of Pará, Brazil (Appendix A).

In 2009 (T0), before the construction of the Belo Monte Dam began, a total of 66 individuals from the Arara-Laranjal (n = 39) and Assurini do Xingu (n = 27) villages were evaluated. The second evaluation was performed 13 years later, in November 2022 (T1), shortly after the construction of the dam was completed. The eligibility criteria considered in T0 were indigenous people in permanent dentition and under 50 years old. At T0, the selected indigenous group had all their permanent teeth. Individuals with craniofacial syndromes or anomalies, such as cleft lip and/or palate, were previously excluded.

### 2.3. Variables, Data Sources, and Measurement

Demographic data such as age and sex were collected. Clinical evaluations of the indigenous participants were carried out by one orthodontist with experience in public health. The evaluation was performed under natural light, with the aid of a flashlight (Petzl, Tikka XP2, France) and disposable tongue depressors. The loss of permanent teeth was the outcome, while sex, age, and village were the predictors. Tooth loss was clinically verified through the presence of prosthetic spaces and by counting the remaining permanent teeth. This count for each individual was made from the right to the left second permanent molars in the upper and lower arches (1.7–2.7 and 3.7–4.7). After applying this method, the total number of missing teeth was calculated for each group (U7: upper second molar; U6: upper first molar; U5: upper second premolar; U4: upper first premolar; U3: upper canine; U2: upper lateral incisor; U1: upper central incisor; L7: lower second molar; L6: lower first molar; L5: lower second premolar; L4: lower first premolar; L3: lower canine; L2: lower lateral incisor; L1: lower central incisor).

### 2.4. Statistical Analysis

Descriptive statistics by village, age, and sex at T0 and T1 were performed. The discrete dependent variable, tooth loss incidence, was analyzed using a multilevel Poisson regression with robust variance. Sex and age were the individual variables included as the first level, and village was the contextual variable. Initially, the association of each predictor variable with the outcome was verified in a bivariate Poisson regression model. In the final model, only the variables that obtained significance in the bivariate analysis were included, considering the *p*-value < 0.1.

No sample size calculation was performed due to the limitation of the population size. Subsequently, a post hoc power analysis was carried out. All statistical analyses were performed using Jamovi software (version 2.3.22, Sydney, Australia) and G*Power (version 3.1, Düsseldorf, Germany), with a significance level of 5%.

## 3. Results

### 3.1. Participants

**Figure 1:**
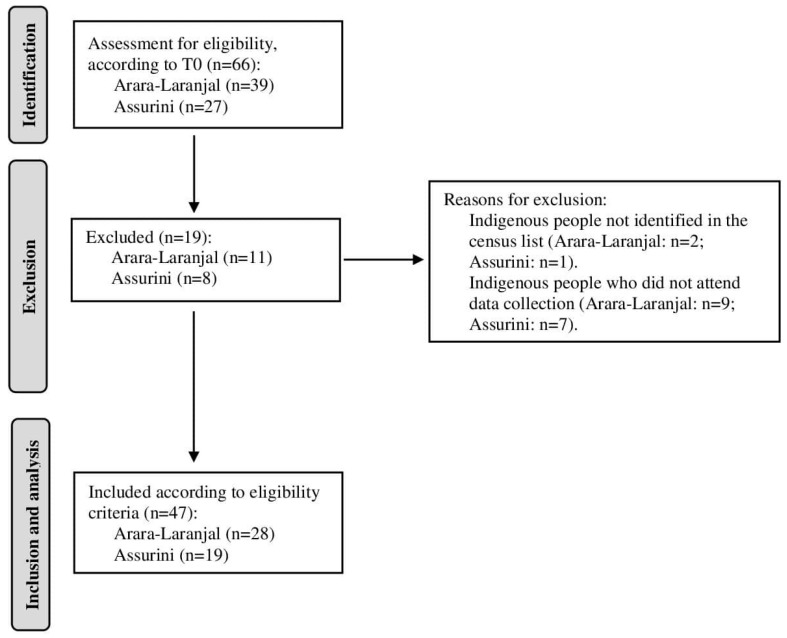
Flow diagram of selected participants from Arara-Laranjal (ethnicity Arara) and Koatinemo (ethnicity Assurini do Xingu).

From the total population previously examined (T0) from Arara (n = 39) and Assurini villages (n = 27), 19 indigenous participants were excluded. According to the eligibility criteria, 47 indigenous participants (71.2% response rate) were included: 28 (71.8%) from Arara and 19 (70.4%) from Assurini (Figure 1).

### 3.2. Descriptive Data

The final sample included a total of 47 indigenous participants: 17 males (36.2%) and 30 females (63.8%). The mean age was similar for both villages at the two evaluation times: 15.9 (±5.67) years old and 16.1 (±4.90) years old at T0, and 29.5 (±5.75) years old and 29.9 (±4.83) years old at T1 in Assurini and Arara villages, respectively (Table 1).

There was an 89% increase in individuals with tooth loss from T0 to T1, as 42 out of 47 indigenous had lost at least one tooth. The incidence of tooth loss was higher (97%) for females compared to males (76%). The total tooth loss was equal to 106 in Arara (median = 4) and 66 in Assurini (median = 3). Ninety-seven teeth were lost in the upper arch and 75 in the lower arch, totaling 172 teeth lost at T1 (median = 3) (Table 1).

**Figure 2:**
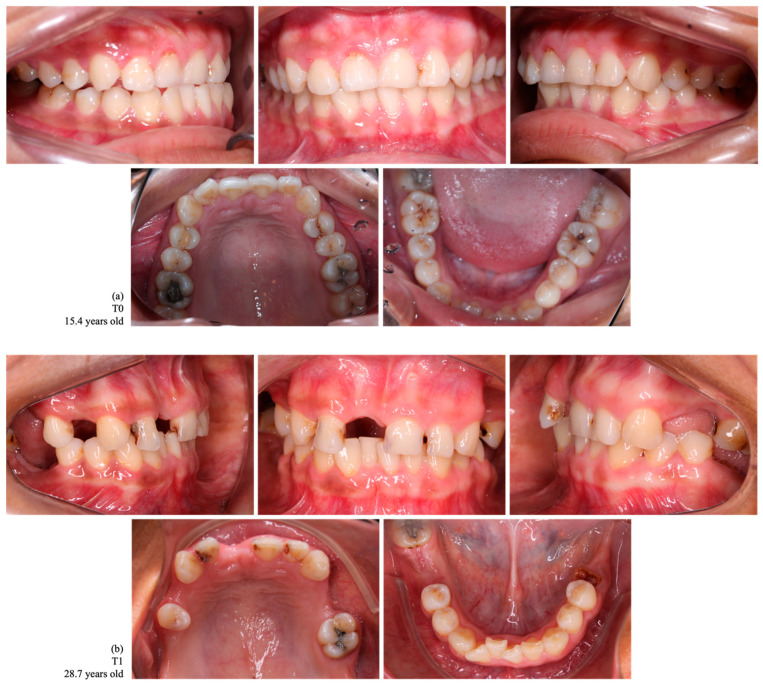
Intraoral photographs of dental occlusion of a female indigenous participant, a habitant of the Assurini village, with no tooth loss at T0 (**a**) and with a loss of 10 permanent teeth at T1 (1.7, 1.6, 1.4, 1.1, 2.4, 2.5, 2.7, 3.7, 3.6, and 4.6) (**b**).

**Figure 3:**
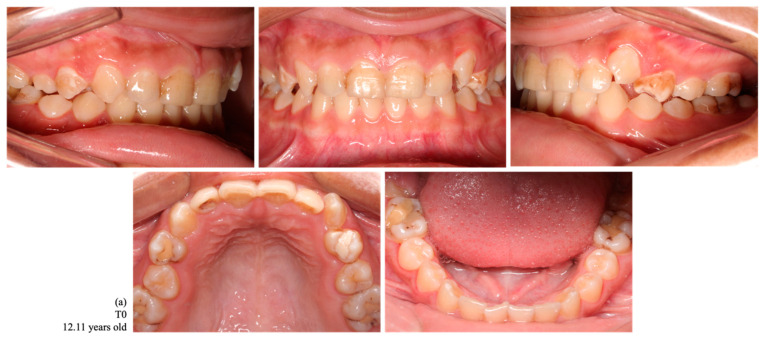

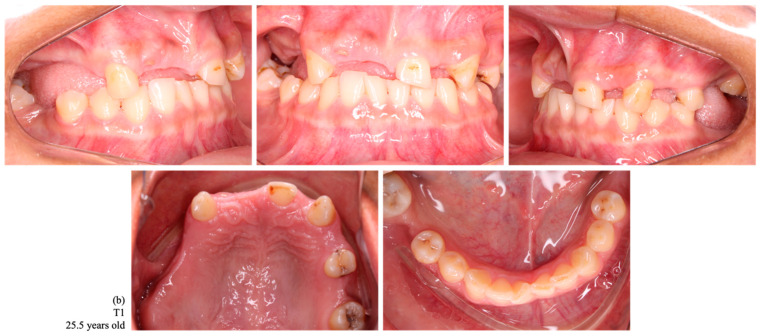
Intraoral photographs of dental occlusion of a female indigenous participant, a habitant of the Arara village, with no tooth loss at T0 (**a**) and with a loss of 12 permanent teeth at T1 (1.7, 1.6, 1.5, 1.4, 1.2, 1.1, 2.2, 2.4, 2.6, 3.7, 3.6, and 4.6) (**b**).

Considering the upper and lower arches, 11 females (23.4%) and seven males (14.9%) lost three or four teeth. Five (10.6%) cases of female indigenous participants recorded more than six teeth lost (Figure 2 and Figure 3). Indigenous participants without tooth loss were found more frequently in males (4/8.5%).

By analyzing the absolute and relative frequency of each group of missing teeth, by initially determining the number of missing teeth in each group through direct counting, the result among females showed that the most affected teeth were the lower second molars (22/46.8%) and upper and lower first molars (17/36.2%). Among males, the highest incidences of tooth loss were in the lower second molars (11/23.4%) and upper first and second molars (6/12.8%) (Table 2).

### 3.3. Main Results

The statistical analysis of the discrete dependent variable, tooth loss, was conducted using a multilevel Poisson regression model. The assumptions of a quantitative positive count variable per unit of time with a quasi-Poisson distribution (tooth loss), a robust variance (variance greater than the mean), the independence of y values (the observations were independent of one another), and linearity (the association between tooth loss and the independent variable must be linear, i.e., *p* < 0.1) were followed. There was no association of the variation in total tooth loss between the villages, verified by the intraclass correlation coefficient equal to zero (ICC = 0.00), which was the indicator of the degree of dependence within the cluster. Moreover, the *p*-value of the likelihood test (LRT) generated by a multilevel linear regression model was not significant (*p* = 1.00). Therefore, there is no evidence of the need to group the villages into separate clusters (Table 3).

In the evaluation of the explanatory variables based on the bivariate regression model, sex showed a significant association with the incidence of tooth loss (*p* = 0.046). The age at T1 was not included in the final model as it did not influence the outcome variable. By fulfilling the assumption of *p* < 0.1, sex was the only variable included in the multilevel Poisson model, thus considering indigenous individuals and villages as the first and second level, respectively. The statistical significance of sex on the total tooth loss was corroborated (*p* = 0.004, 95% CI 0.43 to 0.85). Furthermore, interpreting the β value (estimate), it can be inferred by the female sex, as the reference level, that for males, there was a 0.50 times lower risk of losing teeth when compared to females.

As this is an incidence study, the measurement of association considered for the exponential β is the relative risk. Thus, the incidence of tooth loss was 0.60 times lower among males when compared to females (Table 3).

A post hoc power analysis was performed based on a multiple regression analysis. The R2 value was 0.087 to calculate the effect size, the α error was 0.05, and the sample size was 47 individuals, modulated by one predictor variable, sex. The power was estimated at 54% (Table 3).

## 4. Discussion

The geographic environment and the degree of social isolation directly impact oral health, demonstrating that more isolated individuals tend to have poorer hygiene conditions [14]. To understand the interaction of these factors on tooth loss, it is important to consider that the Amazon is experiencing a drastic socioeconomic situation. This situation is particularly related to the following: lack of water fluoridation and sewage services, less than ideal oral hygiene habits, restricted access to dental services and preventive programs, and incorporation of highly cariogenic foods into their daily routine with the history of recent contact with urban populations [9,15].

The construction of the Belo Monte Hydroelectric Dam led to substantial social and environmental inequities, including displacing at least 20,000 people, including indigenous groups [8,16]. Additionally, the arrival of thousands of people during the construction of the dam may have impacted the remote indigenous population due to their proximity to the urban environment. All these social and economic impacts probably contributed to the change in dietary patterns observed over recent years [2,3,4]. Additionally, it may justify the absence of 16 indigenous participants who did not attend the exam in the villages due to the progressive displacement and the continuous process of fission into smaller groups [7,8].

The main objective of this prospective cohort study was to evaluate the incidence of tooth loss and associated factors among semi-isolated indigenous populations from the Medium Valley of the Xingu River over a time span of 13 years. In 2009, considering a sample of 102 indigenous participants from both villages, the prevalence of tooth loss was 37% (n = 38), totaling 84 teeth. As expected, there was a higher tooth loss from T0 to T1, and the incidence of tooth loss in the sample of 47 indigenous participants analyzed was 89%. Increasing age is considered a possible associated factor with an increased incidence of tooth decay and, consequently, tooth loss [9,17,18,19].

In an indigenous population from Northeast Brazil, the prevalence of teeth with extraction indication due to carious lesions was 4.98%, characterizing an average of 1.24 teeth per individual [19]. Globally, the prevalence and severity of tooth decay are higher among indigenous compared to non-indigenous groups, regardless of age, gender, or country, which can be associated with poorer oral hygiene habits [20,21]. Particularly among urban and rural indigenous populations, the last-mentioned presented significantly more missing teeth [22]. The highest rates are particularly observed for individuals with untreated dental decay and teeth already lost [20,21]. In addition to the pathological factor related to tooth loss, data from the indigenous populations of the Amazon region revealed injury and handmade activity as an etiological factor for tooth loss [23].

In the multilevel Poisson regression analysis, the results indicated a lower risk and incidence of tooth loss for males when compared to females, which is a sex reversal situation compared to the larger sample in 2009, as 27% of females and 47% of males lost at least one tooth. The hormonal fluctuations experienced by women make the oral environment significantly more cariogenic for them [24]. However, the cultural factor related to the social functions of each sex and the different forms of access to information, health services, and education must also be considered [25]. In accordance with other studies [11,26], females also experienced a greater risk of tooth loss related to a higher amount of caries. It can be inferred that females tend to self-care and use oral health services more frequently, which leads to overtreatment and consequent early tooth loss. In the division of labor, males are often away from home, serving in subsistence activities such as hunting, fishing, and war clashes [27], while females have greater access to food [28]. Differently, other studies indicated that males were more subject to tooth loss [29,30]. Therefore, the finding of sexual dimorphism in relation to tooth loss is still controversial in the literature.

In relation to the dental arch affected by tooth loss, this study observed 97 tooth losses in the maxilla and 75 in the mandible, totaling 172 tooth losses after 13 years. In another cohort study, with a 4-year follow-up period, the prevalence of tooth loss was higher in the maxilla at baseline (53.7%), and the incidence was similar between the upper (50.8%) and the lower (49.2%) arches in the follow-up period, with a total of 130 teeth lost among 51 adults. However, this result should be interpreted with caution in comparison to the findings of the present study as the evaluation was carried out in an older urban population, and for calculating the cumulative incidence of tooth loss, 32 teeth were considered [17].

The most frequently lost teeth were first and second molars. Findings from other studies also pointed to first molars as the most commonly missing teeth [17,26,31,32].

The multilevel Poisson regression demonstrated there was no association between the total tooth loss and the villages. This probably occurred due to the semi-isolated Amazon indigenous people being subject to the same environmental conditions characterized by traditional eating habits [5]. In addition to the environmental factor involved in the etiology of dental caries, individual susceptibility also depends on preventive factors for caries disease, including genetics—the biochemical composition of saliva and overall saliva flow rate—which can counteract the negative eating habits of individuals [24,33].

In this context, dental caries and periodontal diseases sound an alarm for an unhealthy diet, and the main outcome, therefore, is the balance between oral health promotion and the offer of protective factors [34]. The parental ability to sustain an inverse trajectory of maintaining traditional cultural habits, above all eating habits, can play an important role in dental caries prevention and, consequently, in the incidence of tooth loss in some indigenous communities, especially among children and adolescents [35]. It must also be considered that both indigenous groups have the same opportunities to access oral health services. This justifies the greater focus on curative assistance instead of health promotion and disease prevention action is, perhaps, the individual and subjective desire of each indigenous. Thus, it is easier to reach the endpoint of oral diseases, which is tooth loss.

### Limitations

The present study has some limitations that should be considered. The majority of participants were females. Furthermore, as in other cohort study designs, there was a loss of sample data in 19 indigenous participants over the 13 years, reducing the sample size by 28.8% (n = 19/66).

Several factors can influence tooth loss. Education, culture, psychological factors, and nutrition, such as a sugar-based diet, influenced the oral health history of the indigenous after the construction of the Belo Monte Dam. However, the influence of these factors on tooth loss has not been thoroughly investigated, leading to the hypothesis that they may have contributed to tooth loss following the construction of the dam. Therefore, the findings of this study must be extrapolated with caution in relation to the urbanization process provided by the construction of the dam.

Although the power analysis was equal to 54%, the sample of this study is considered limited due to its convenience selection, and there was no impact on the result of the regression model.

Finally, the results of this study are of importance for understanding tooth loss in a traditional population. New studies are necessary to better understand the etiology of tooth loss in indigenous people. The results support the creation of effective and specific oral health programs in order to protect these traditional populations from the damage of dental disease, early tooth loss, and its aesthetic and functional consequences, as well as improve their oral health and quality of life.

## 5. Conclusions

A high incidence of tooth loss in remote indigenous populations living in the Amazon region was observed. The incidence of tooth loss is not different between the indigenous villages examined, and it occurs similarly in younger and older indigenous people. Female indigenous participants had two times more tooth loss compared to males. Additionally, the first and second permanent molars were the most affected teeth. These results suggest that the construction of the Belo Monte Dam may have influenced this high incidence of tooth loss. Immediate public health policies to protect traditional populations by providing oral health care are mandatory, especially among females.

## Figures and Tables

**Table 1:** Descriptive statistics. Assessment by village (n), sex (male M/female F), mean (X), and standard deviation (±SD) of age at T0 and T1, total, median, and first and third quartiles (Q1–Q3) of tooth loss in the upper, lower and both arches.

Variables		Village		
		Assurini (n = 19)	Arara-Laranjal (n = 28)	Total (n = 47)
Sex (M/F)		8/11	9/19	
Age T0 X(±SD)		15.9 (±5.67)	16.1 (±4.90)	
Age T1 X(±SD)		29.5 (±5.75)	29.9 (±4.83)	
Total tooth loss T1-T0 (median/Q1-Q3)	Upper arch	34 (1/0.5–2.5)	63 (2/0–3)	97 (2/0–3)
	Lower arch	32 (2/1–2)	43 (1/1–2.25)	75 (2/1–2)
	Total	66 (3/2–4.5)	106 (4/1–5.25)	172 (3/2–5)

**Table 2:** Absolute and relative frequency related to each group of tooth loss, according to the village and sex (male M/female F).

		Sex	Village				Sex	Village		
			Assurini (n = 19)	Arara-Laranjal (n = 28)	Both Villages (n = 47)			Assurini (n = 19)	Arara-Laranjal (n = 28)	Both Villages (n = 47)
Number of tooth loss in both arches (n/%)	U7	M	3/15.8%	3/10.7%	6/12.8%	L7	M	6/31.6 %	5/17.9%	11/23.4%
		F	4/21.1%	6/21.4%	10/21.3%		F	8/42.1%	14/50.0%	22/46.8%
	U6	M	2/10.5%	4/14.3%	6/12.8%	L6	M	1/5.3%	2/7.1%	3/6.4%
		F	6/31.6 %	11/39.3%	17/36.2%		F	8/42.1%	9/32.1%	17/36.2%
	U5	M	1/5.3%	1/3.6%	2/4.3%	L5	M	0/0.0%	1/3.6%	1/2.1%
		F	4/21.1%	7/25.0%	11/23.4%		F	0/0.0%	1/3.6%	1/2.1%
	U4	M	2/10.5%	0/0.0%	2/4.3%	L4	M	0/0.0%	0/0.0%	0/0.0%
		F	5/26.3%	8/28.6%	13/27.7%		F	0/0.0%	0/0.0%	0/0.0%
	U3	M	0/0.0%	0/0.0%	0/0.0%	L3	M	0/0.0%	0/0.0%	0/0.0%
		F	0/0.0%	0/0.0%	0/0.0%		F	0/0.0%	0/0.0%	0/0.0%
	U2	M	1/5.3%	1/3.6%	2/4.3%	L2	M	0/0.0%	0/0.0%	0/0.0%
		F	0/0.0%	3/10.7%	3/6.4%		F	0/0.0%	0/0.0%	0/0.0%
	U1	M	0/0.0%	0/0.0%	0/0.0%	L1	M	0/0.0%	0/0.0%	0/0.0%
		F	1/5.3%	3/10.7%	4/8.5%		F	0/0.0%	0/0.0%	0/0.0%

U7: upper second molar; U6: upper first molar; U5: upper second premolar; U4: upper first premolar; U3: upper canine; U2: upper lateral incisor; U1: upper central incisor; L7: lower second molar; L6: lower first molar; L5: lower second premolar; L4: lower first premolar; L3: lower canine; L2: lower lateral incisor; L1: lower central incisor.

**Table 3:** Multilevel Poisson regression (level 1 individuals, level 2 villages) for the association between the predictor variable sex and the increase in total tooth loss in the upper and lower arches (dependent variable). R2 = 0.087.

Independent Variables	Bivariate Model			Multilevel Poisson Regression							Sample Power
	*p*-Value	CI (95%)		*p*-Value	Estimate	Exp(β)	CI (95%)		ICC	*p*-Value (LRT)	
		Lower	Upper				Lower	Upper			
(Village)									0.00	1.00	0.54 (54%)
Sex (M-F)	0.046 *	0.37	0.96	0.004 **	−0.50	0.60	0.43	0.85			
Age in T1	0.590	0.94	1.03								

M: male; F: female; CI: confidence interval; ICC: intraclass correlation coefficient; LRT: likelihood test. * Statistically significant at *p* < 0.10; ** Statistically significant at *p* < 0.05.

## Data Availability

The data presented in this study are available upon reasonable request from the corresponding author.

## Appendix A

Previously published studies carried out in indigenous populations located on the region of the Medium Valley of the Xingu River, State of Pará, Brazil.

1. Normando, D.; Santos, H.G.A.; Quintão, C.C.A. Comparisons of tooth sizes, dental arch dimensions, tooth wear, and dental crowding in Amazonian indigenous people. *Am. J. Orthod. Dentofac. Orthop.* **2016**, *150*, 839–846. https://doi.org/10.1016/j.ajodo.2016.03.033
2. Normando, D.; Almeida, M.A.; Quintão, C.C. Dental crowding: the role of genetics and tooth wear. *Angle Orthod.* **2013**, *83*, 10–15. https://doi.org/10.2319/020112-91.1
3. Normando, D.; Faber, J.; Guerreiro, J.F.; Abdo Quintão, C.C. Dental occlusion in a split Amazon indigenous population: genetics prevails over environment. *PLoS ONE* **2011**, *6*, e28387. https://doi.org/10.1371/journal.pone.0028387
4. Vieira, E.P.; Barbosa, M.S.; Quintão, C.C.; Normando, D. Relationship of tooth wear to chronological age among indigenous Amazon populations. *PLoS ONE* **2015**, *10*, e0116138. https://doi.org/10.1371/journal.pone.0116138
5. Bastos, R.T.D.R.M.; Neto, J.V.; Normando, D. Dentofacial biometry as a discriminant factor in the identification of remote Amazon indigenous populations. *Am. J. Orthod. Dentofac. Orthop.* **2020**, *157*, 619–630. https://doi.org/10.1016/j.ajodo.2019.05.016
6. de Souza, B.S.; Bichara, L.M.; Guerreiro, J.F.; Quintão, C.C.; Normando, D. Occlusal and facial features in Amazon indigenous: An insight into the role of genetics and environment in the etiology dental malocclusion. *Arch. Oral Biol.* **2015**, *60*, 1177–1186. https://doi.org/10.1016/j.archoralbio.2015.04.007
7. Barbosa, M.; Vieira, E.P.; Quintão, C.C.; Normando, D. Facial biometry of Amazon indigenous people of the Xingu River—Perspectives on genetic and environmental contributions to variation in human facial morphology. *Orthod. Craniofac. Res.* **2016**, *19*, 169–179. https://doi.org/10.1111/ocr.12125
8. Normando, D.; Barbosa, M.S.; Mecenas, P.; Quintão, C. Tooth wear as an indicator of acculturation process in remote Amazonian populations. *PLoS ONE* **2020**, *15*, e0230809. https://doi.org/10.1371/journal.pone.0230809
